# Clinical implication of the advanced lung cancer inflammation index in patients with right-sided colon cancer after complete mesocolic excision: a propensity score-matched analysis

**DOI:** 10.1186/s12957-022-02712-0

**Published:** 2022-08-01

**Authors:** Yu Deng, Yanwu Sun, Yu Lin, Ying Huang, Pan Chi

**Affiliations:** Department of Colorectal Surgery, Fujian Medical University Union Hospital, 29 Xinquan Road, Fuzhou, Fujian 350001 People’s Republic of China

**Keywords:** Advanced lung cancer inflammation index, Right-sided colon cancer, Postoperative complications, Prognosis, Propensity score-matched

## Abstract

**Background:**

This study aimed to assess the clinical implications of the advanced lung cancer inflammation index (ALI) in patients with right-sided colon cancer (RCC) after complete mesocolic excision (CME).

**Methods:**

A total of 441 patients with RCC who underwent CME were included. The optimal cut-off value for the ALI was determined using the X-tile software. Logistic and Cox regression analyses were used to identify risk factors for postoperative complications and long-term outcomes. Predictive nomograms for overall survival (OS) and disease-free survival (DFS) were constructed after propensity score matching (PSM), and their performance was assessed using the net reclassification improvement index (NRI), integrated discrimination improvement index (IDI), and time-dependent receiver operating characteristic (time-ROC) curve analysis.

**Results:**

The optimal preoperative ALI cut-off value was 36.3. After PSM, ASA classification 3/4, operative duration, and a low ALI were independently associated with postoperative complications in the multivariate analysis (all *P*<0.05). Cox regression analysis revealed that an age >60 years, a carbohydrate antigen 19-9 (CA19-9) level >37 U/mL, pathological N+ stage, and a low ALI were independently correlated with OS (all *P*<0.05). A CA19-9 level >37 U/mL, pathological N+ stage, lymphovascular invasion, and a low ALI were independent predictors of DFS (all *P*<0.05). Predictive nomograms for OS and DFS were constructed using PSM. Furthermore, a nomogram combined with the ALI was consistently superior to a non-ALI nomogram or the pathological tumor-node-metastasis classification based on the NRI, IDI, and time-ROC curve analysis after PSM (all *P*<0.05).

**Conclusion:**

The ALI was an effective indicator for predicting short- and long-term outcomes in patients with RCC.

**Supplementary Information:**

The online version contains supplementary material available at 10.1186/s12957-022-02712-0.

## Background

Colon cancer, one of the most common cancers, is the third most common cause of cancer-related deaths worldwide [1]. Right-sided colon cancer (RCC) is a distinct entity in terms of its anatomy, biology, and prognosis compared with left-sided colon cancer [2]. Complete mesocolic excision (CME) with D3 lymph node dissection is the surgical principle for RCC that can improve surgical quality and, thus, oncological outcomes [3, 4]. However, tumor recurrence and metastasis remain significant factors that contribute to poor patient survival [5]. Systemic inflammation is closely associated with tumor development and progress [6]. Nutritional indicators are also associated with the prognosis of various malignancies [7, 8]. The advanced lung cancer inflammation index (ALI), a novel inflammation and nutrition-based index defined by combining body the mass index (BMI), preoperative serum albumin (ALB) level, and neutrophil-to-lymphocyte ratio (NLR), has been proposed as a prognostic biomarker for various malignant tumors, including lung, esophageal, gastric, and colorectal cancers [9–14]. In colon cancer, patients with RCC tend to experience malnutrition before surgery, leading to immune depression [15]. No large-scale study has evaluated the clinical implications of preoperative ALI in patients with RCC yet.

The American Joint Committee on Cancer (AJCC) staging system is most commonly used to assess the prognosis of cancer patients [16]. The current tumor-node-metastasis (TNM) framework relies exclusively on locoregional tumor expansion(s) of the primary tumor but neglects substantial tumor- and host-related biological differences [17]. However, survival may differ in patients with RCC, even among those with the same TNM stage. Therefore, combining the AJCC/TNM staging system with other prognostic indicators may improve the individual prognosis prediction of patients with RCC and facilitate treatment decision-making.

In this context, the present study aimed to explore the prognostic value of the ALI and to establish a simple scoring system based on the ALI to effectively predict the short- and long-term outcomes of patients with RCC after CME.

## Methods

### Patients

A total of 441 consecutive patients who underwent CME with D3 lymph node dissection for RCC between January 2012 and December 2016 at our department were included. Clinicopathological data were collected from the colorectal cancer database. The inclusion criteria were as follows: colon adenocarcinoma; tumors in the ileocecum, ascending colon, or right colic flexure; and pathological stage I–III disease. Individuals who underwent emergency surgery, had synchronous or metachronous multiple primary colorectal cancer(s), and those with incomplete clinicopathological data were excluded.

### Definitions, surgical procedures, adjuvant chemotherapy, and follow-up

All laboratory data were obtained within 2 weeks before surgery. Preoperative anemia was defined as a hemoglobin level < 120 g/L. The formulae for calculating the ALI, systemic inflammation index (SII) [18], and prognostic nutritional index (PNI) [18] were as follows: ALI= BMI × albumin/NLR; SII = platelet (10^11^/L) × NLR; PNI = preoperative serum albumin (g/L) + 5 × total preoperative lymphocyte count (10^9^/L). All patients underwent radical surgery following the principle of CME [19] with D3 lymph node dissection. Approximately 4 to 8 weeks after radical resection, patients with high-risk stage II and stage III disease underwent four to eight cycles of 5-fluorouracil-based adjuvant chemotherapy for 3 to 6 months, including the “XELOX” and “FOLFOX” regimens. Postoperative surveillance was conducted every 3 months for the first 2 years, twice per year for the next 3 years, and once per year thereafter, including serum carcinoembryonic antigen (CEA), chest computed tomography (CT), and abdominopelvic magnetic resonance imaging (MRI) or CT. Colonoscopy was performed 3 months to 1 year after surgery and once every year thereafter. Follow-up information was obtained through clinics or telephonic interviews. Information regarding patients lost to follow-up was obtained from the Chinese Population Registration and Health Insurance System. Patient follow-up was performed until death or April 1, 2021.

### Statistical analysis

Statistical analyses were performed using SPSS version 20.0 (IBM SPSS Statistics, Chicago, IL, USA) and the R software (version 3.6.1). The X-tile software [20] was used to identify the optimal cut-off values for the ALI, PNI, and SII according to 5-year overall survival (OS). To minimize the biasing effects of confounders, a 1:1 caliper width of 0.2 for the propensity score matching (PSM) analysis was performed on the following variables: age, sex, preoperative CEA and carbohydrate antigen 19-9 (CA19-9) levels, pT stage, pN stage, pTNM stage, histological tumor differentiation, nerval invasion (NI), and lymphovascular invasion (LVI). Pairs of patients were selected using the “nearest-neighbor” matching method. The chi-squared test or Fisher’s exact test was used to compare categorical variables, and the Student’s *t* test was used to compare continuous variables. Logistic regression analyses were performed to identify independent risk factors for postoperative complications. Cox proportional hazard models were used to evaluate the prognostic value of the ALI. The prognostic efficacy of different models was assessed according to the net reclassification improvement index (NRI) [21, 22], integrated discrimination improvement index (IDI) [21, 22], and time-dependent receiver operating characteristic (time-ROC) curve analysis. Differences with *P*<0.05 were considered to be statistically significant.

## Results

### Patient characteristics

Among the 441 patients included in this study, 236 (53.5%) and 205 (46.5%) were classified as having a low and high ALI, respectively, based on the ALI cut-off value of 36.3 for 5-year OS according to the X-tile program (Supplementary Figure 1A). Similarly, 262 (59.4%) and 179 (40.6%) patients were divided into low and high PNI groups (cut-off value of 48 for 5-year OS) (Supplementary Figure 1B), respectively, while 165 (37.4%) and 276 (62.6%) patients were included in the low and high SII groups (cut-off value of 427 for 5-year OS) (Supplementary Figure 1C), respectively. The predictive ability of the ALI for 5-year OS was better than that of the PNI (area under curve (AUC) 0.644 [95% CI 58.90–69.88] vs. 0.600 [95% CI 52.36–63.72]; *P*<0.001) or SII (AUC 0.644 [95% CI 58.90–69.88] vs. 0.580 [95% CI 54.62–65.40]; *P*<0.001) (Supplementary Figure 2A). The ALI was superior to the PNI (AUC 0.609 [95% CI 55.02–66.90] vs. 0.556 [95% CI 49.49–61.61]; *P*=0.014) and SII (AUC 0.609 [95% CI 55.02–66.90] vs. 0.555 [95% CI 49.66–61.50]; *P*=0.005) in predicting 5-year disease-free survival (DFS) (Supplementary Figure 2B).

The clinicopathological characteristics of all patients with RCC are summarized in Table 1. After 1:1 PSM, 180 paired patients were matched to the low and high ALI groups. Most covariates were well balanced, except for preoperative anemia (*P*=0.003), surgical approach (*P*=0.004), and postoperative hospital stay (*P*=0.014).

**Table 1:** Baseline characteristics of right-sided colon cancer patients with preoperative low and high ALI.

Characteristics	Before PSM				After PSM			
	Total (*n* = 441)	Low-ALI	High-ALI	*P* value	Total (*n* = 360)	Low-ALI	High-ALI	*P* value
		(*n* = 236)	(*n* = 205)			(*n* = 180)	(*n* = 180)	
Age, n (%)				0.015				0.399
≤60 years	207 (46.9)	98 (41.5)	109 (53.2)		186 (51.7)	89 (49.4)	97 (53.9)	
>60 years	234 (53.1)	138 (58.5)	96 (46.8)		174 (48.3)	91 (50.6)	83 (46.1)	
Gender, n (%)				0.815				0.672
Male	234 (53.1)	124 (52.5)	110 (53.7)		194 (53.9)	95 (52.8)	99 (55.0)	
Female	207 (46.9)	112 (47.5)	95 (46.3)		166 (46.1)	85 (47.2)	81 (45.0)	
ASA classification, n (%)				0.203				0.115
1+2	410 (93.0)	216 91.5)	194 (94.6)		332 (92.2)	162 (90.0)	170 (94.4)	
3+4	31 (7.0)	20 (8.5)	11 (5.4)		28 (7.8)	18 (10.0)	10 (5.6)	
Preoperative anemia	275 (62.4)	165 (69.9)	110 (53.7)	<0.001	223 (61.9)	125 (69.4)	98 (54.4)	0.003
Preoperative CEA, n (%)				0.430				0.748
≤5 ng/ml	271 (61.5)	141 (59.7)	130 (63.4)		213 (59.2)	105 (58.3)	108 (60.0)	
>5 ng/ml	170 (38.5)	95 (40.3)	75 (36.6)		147 (40.8)	75 (41.7)	72 (40.0)	
Preoperative CA19-9, n (%)				0.119				0.895
≤37 U/ml	347 (78.7)	179 (75.8)	168 (82.0)		289 (78.7)	145 (80.6)	144 (80.0)	
>37 U/ml	94 (21.3)	57 (24.2)	37 (18.0)		71 (21.3)	35 (19.4)	36 (20.0)	
Diabetes, n (%)	101 (15.3)	7 (14.6)	94 (15.3)	0.889	53 (14.7)	28 (15.6)	25 (13.9)	0.655
Hypertension, n (%)	186 (28.1)	14 (29.2)	172 (28.1)	0.869	102 (28.3)	47 (26.1)	55 (30.6)	0.349
Surgical approach, n (%)				0.001				0.004
Laparoscopic	317 (71.9)	154 (65.3)	163 (79.5)		255 (29.2)	115 (63.9)	140 (77.8)	
Open	124 (28.1)	82 (34.7)	42 (20.5)		105 (70.8)	65 (36.1)	40 (22.2)	
Tumor location, n (%)				0.242				0.598
Ileocecal /ascending colon	217 (49.2)	110 (46.6)	107 (52.2)		179 (49.7)	87 (48.3)	92 (51.1)	
Hepatic flexure colon	224 (50.8)	126 (53.4)	98 (47.8)		181 (50.3)	93 (51.7)	88 (48.9)	
Total retrieved LNs				0.404				0.723 ^a^
<12	12 (2.7)	5 (2.1)	7 (3.4)		8 (2.2%)	3 (1.7%)	5 (2.8%)	
≥12	429 (97.3)	231 (97.9)	198 (96.6)		352 (97.8%)	177 (98.3%)	175 (97.2%)	
Operative time, n (%)				0.527				0.398
≤205 min	208 (47.2)	108 (45.8)	100 (48.8)		166 (46.1)	79 (43.9)	87 (48.3)	
>205 min	233 (52.8)	128 (54.9)	105 (51.2)		194 (53.9)	101 (56.1)	93 (51.7)	
Estimated blood loss, n (%)				0.221				0.292
≤55 ml	225 (51.0)	114 (48.3)	111 (54.1)		182 (50.6)	86 (47.3)	96 (53.3)	
>55 ml	216 (49.0)	122 (51.7)	94 (45.9)		178 (49.4)	94 (52.2)	84 (46.7)	
pT stage, n (%)				<0.001				0.684
T1+2	48 (10.9)	12 (5.1)	36 (17.6)		26 (10.9)	12 (6.7)	14 (7.8)	
T3+4	393 (89.1)	224 (94.9)	169 (82.4)		334 (89.1)	168 (93.3)	166 (92.2)	
pN stage, n (%)				0.413				0.590
N0	248 (56.2)	128 (54.2)	120 (58.5)		205 (56.9)	100 (55.6)	105 (58.3)	
N+	193 (43.8)	108 (45.8)	85 (44.3)		155 (43.1)	80 (44.4)	75 (41.7)	
pTNM stage, n (%)				0.004				0.762
I	39 (9.5)	11 (8.3)	28 (9.6)		25 (9.5)	11 (6.1)	14 (7.8)	
II	209 (46.5)	117 (54.2)	92 (45.7)		180 (46.5)	89 (49.4)	91 (50.6)	
III	193 (44.0)	108 (37.5)	85 (44.7)		155 (44.0)	80 (44.5)	75 (41.7)	
Tumor differentiation, n (%)				0.014				0.673
Grade 1+2	399 (90.5)	206 (87.3)	193 (94.1)		336 (93.3)	167 (92.8)	169 (93.9)	
Grade 3+4	42 (9.5)	30 (12.7)	12 (5.9)		24 (6.7)	13 (7.2)	11 (6.1)	
Histopathology, n (%)				0.960				0.521
Adenocarcinoma	263 (59.6)	141 (59.7)	122 (59.5)		210 (90.5)	108 (60.0)	102 (56.7)	
Mucinous/signet ring cell adenocarcinoma	178 (40.4)	95 (40.3)	83 (40.5)		150 (9.5)	72 (40.0)	78 (43.3)	
Nerval invasion, n (%)	46 (16.3)	26 (11.0)	20 (9.8)	0.666	36 (10.0)	17 (9.4)	19 (10.6)	0.725
Lymphovascular invasion, n (%)	67 (19.2)	40 (16.9)	27 (13.2)	0.270	48 (13.3)	24 (13.3)	24 (13.3)	0.270
Adjuvant chemotherapy	295 (66.9)	165 (69.9)	130 (63.4)	0.148	242 (67.2)	123 (68.3)	119 (66.1)	0.653
Postoperative hospital stays, mean (SD)	8.88 (5.35)	9.61 (5.44)	8.23 (5.47)	0.009	8.82 (5.30)	9.53 (4.97)	8.14 (5.528)	0.014

^a^: Fisher’s exact test.

*PSM* propensity score match, *ASA* American Society of Anesthesiologists, *ALI* advanced lung cancer inflammation index, *CEA* carcinoembryonic antigen, *CA19-9* carbohydrate antigen 19-9, *LNs* lymph nodes, *SD* standard deviation.

### Correlation between the ALI and postoperative morbidity

A total of 114 (25.9%) patients developed postoperative complications before PSM, with the majority classified as having Clavien-Dindo classification grades II–III, among whom 23 experienced ≥ 2 complications. The most frequent morbidity was pneumonia (12.5% [*n*=55]), followed by chylous ascites (10.2% [*n*=45]), urinary tract infection (3.4% [*n*=15]), postoperative ileus (2.5% [*n*=11]), surgical site infection (2.0% [*n*=9]), intra-abdominal infection (1.6% [*n*=7]), delayed gastric emptying (1.1% [*n*=5]), intra-abdominal bleeding (0.5% [*n*=1]), and septicemia (0.2% [*n*=1]). No complication-related deaths occurred.

After PSM, the American Society of Anesthesiologists (ASA) classification 3/4 (odds ratio [OR]: 2.843, [95% CI 1.276–6.334]; *P*=0.011), operative duration (OR 1.723, *P*=0.032), and a low ALI (OR 1.983, *P*=0.007) were found to be independent predictors of postoperative complications (Table 2).

**Table 2:** Logistic analysis for postoperative complications in right-sided colon cancer patients in the PSM cohort

Variables	After PSM			
	Univariate		Multivariate	
	OR (95%CI)	*P* value	OR (95%CI)	*P* value
Age (>60 vs. ≤60, years)	1.265 (0.789-2.030)	0.330		
Gender (male vs. female)	0.884 (0.551-1.418)	0.609		
ASA classification (3/4 vs. 1/2)	3.203 (1.464-7.005)	**0.004**	2.843 (1.276-6.334)	**0.011**
Preoperative anemia (<120 vs. ≥120, g/l)	1.404 (0.853-2.313)	0.182		
Preoperative CEA (>5 vs. ≤5, ng/ml)	1.198 (0.743-1.930)	0.459		
Preoperative CA19-9 (>37 vs. ≤37, U/ml)	0.586 (0.304-1.127)	0.109		
Diabetes	1.036 (0.534-2.009)	0.917		
Hypertension	1.047 (0.622-1.764)	0.862		
Surgery access (open vs. laparoscopic)	1.164 (0.686-1.973)	0.574		
Tumor location (ileocecal/ascending colon vs. hepatic flexure colon)	1.280 (0.797-2.055)	0.308		
Operative time (>205 vs. ≤205, min)	1.805 (1.108-2.939)	**0.018**	1.723 (1.047-2.836)	**0.032**
Estimated blood loss (>55 vs. ≤55, ml)	1.597 (0.991-2.573)	0.054		
pT stage (T3/4 vs. T1/2)	0.941 (0.382-2.317)	0.895		
pN stage (N+ vs. N0)	1.122 (0.698-1.805)	0.634		
Tumor differentiation (grade 3+4 vs. 1+2)	0.555 (0.185-1.668)	0.294		
Histopathology (mucinous/signet ring cell adenocarcinoma vs. adenocarcinoma)	1.143 (0.710-1.841)	0.583		
ALI (low vs. high)	2.094 (1.288-3.404)	**0.003**	1.983 (1.209-3.252)	**0.007**

*HR* hazard ratio, *CI* confidence interval, *PSM* propensity score match, *ASA* American Society of Anesthesiologists, *ALI* advanced lung cancer inflammation index, *CEA* carcinoembryonic antigen, *CA19-9* carbohydrate antigen 19-9

### Correlations between the ALI and survival outcomes in the non-PSM and PSM cohorts

The median follow-up duration was 65 months (range, 3–110 months). Before PSM, the 5-year OS rates in low and high ALI patients were 71.0% and 90.5%, respectively. The 5-year cumulative recurrence rates in patients with low and high ALI were 26.7% and 11.5%, respectively. A low ALI was significantly associated with poor 5-year OS (*P*<0.001) (Supplementary Figure 3A) and DFS (*P*<0.001) (Supplementary Figure 3B).

**Fig. 1:**
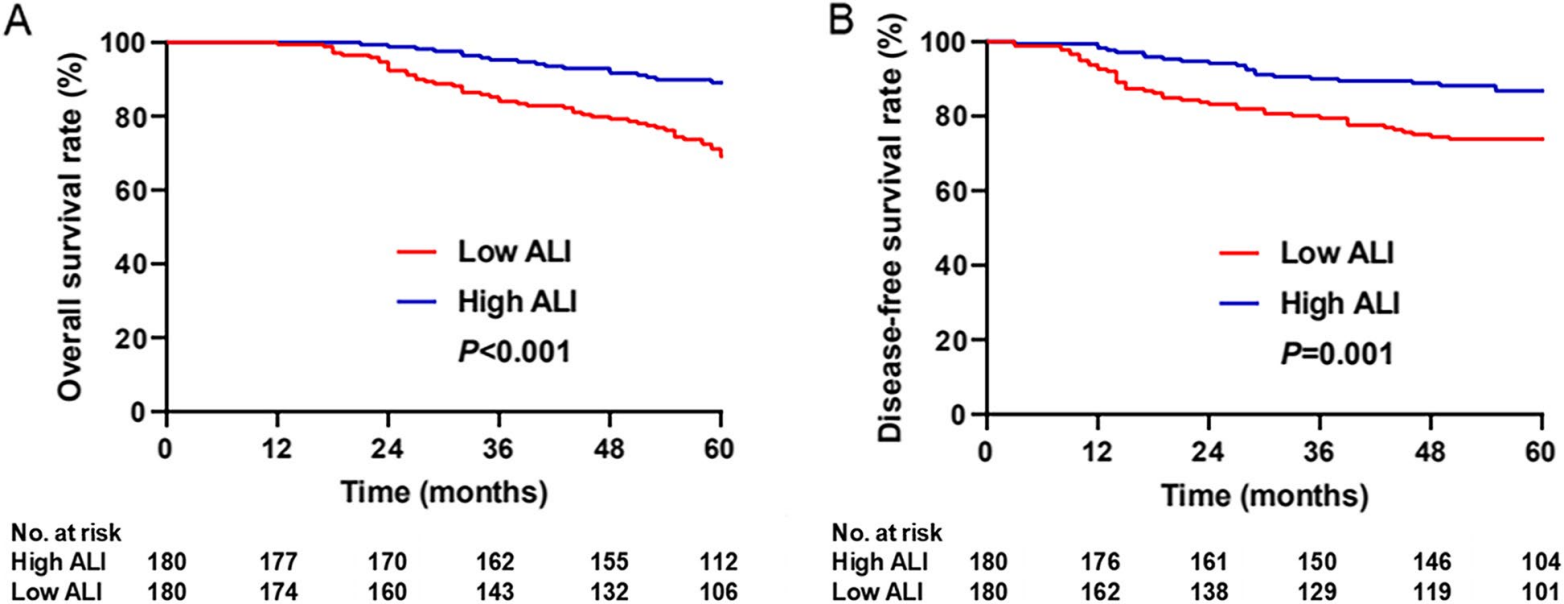
Kaplan-Meier survival analysis according to ALI status in patients with right sided colon cancer. **A** Overall survival of propensity-matched patients. **B** Disease-free survival of propensity-matched patients

**Fig. 2:**
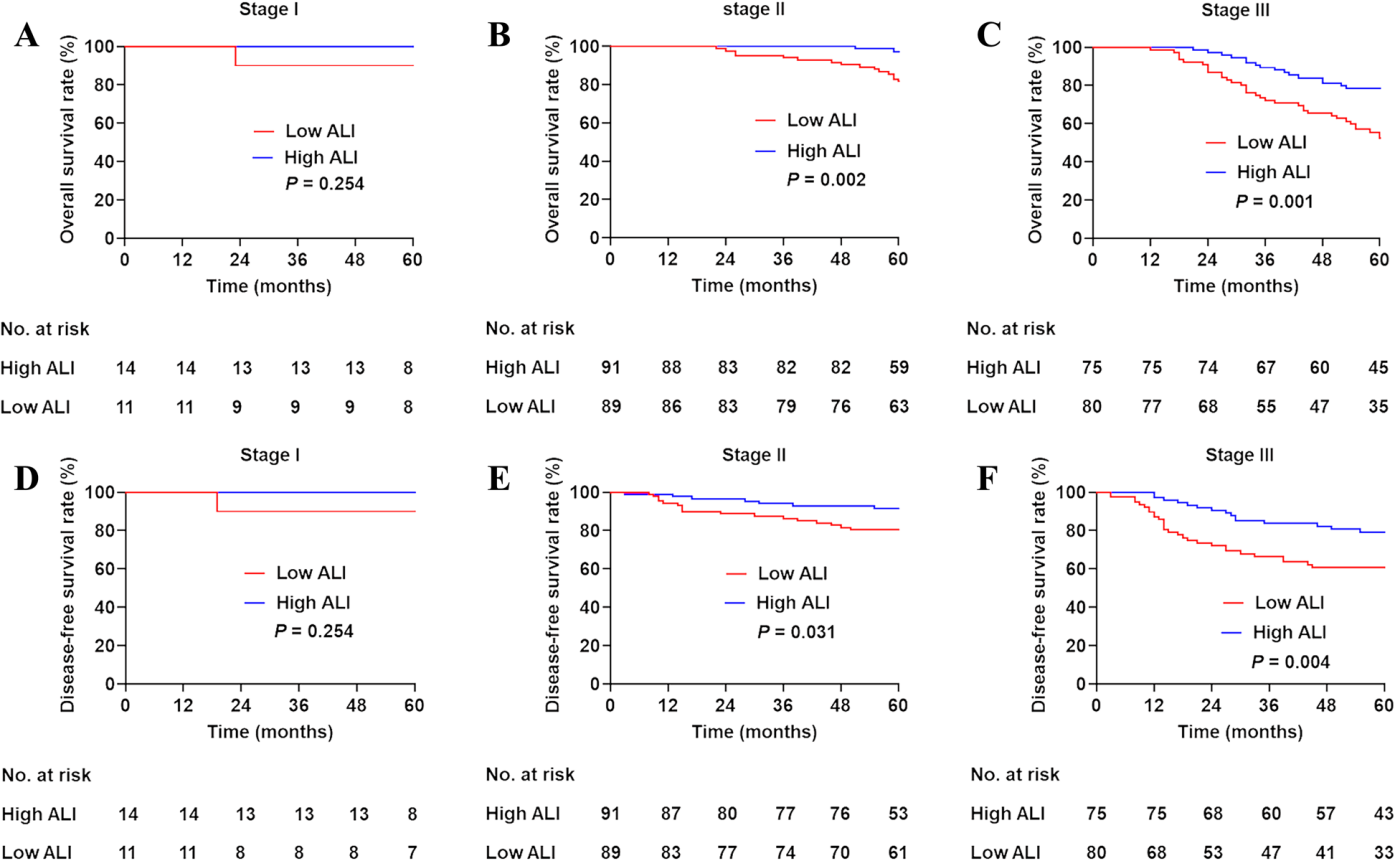
Kaplan-Meier survival analysis according to ALI status in patients with different stages. Overall survival: **A** Stage I, **B** Stage II, and **C** Stage III. Disease-free survival: **D** Stage I, **E** Stage II, and **F** Stage III

After PSM, the 5-year OS rates in low and high ALI patients were 69.1% and 89.1%, respectively. The 5-year cumulative recurrence rates in patients with a low and high ALI were 26.9% and 13.2%, respectively. Patients with a low ALI had worse 5-year OS (*P*<0.001; Fig. 1A) and DFS (*P*=0.001; Fig. 1B) than those with a high ALI. Predictive values of the ALI at different stages were compared. In stage I patients, the 5-year OS was comparable between the high and low ALI groups (*P*=0.254; Fig. 2A). Patients with a low ALI had a worse 5-year OS than those with a high ALI in stage II (*P*=0.02; Fig. 2B) and stage III (*P*=0.01; Fig. 2C). There was no significant difference in the 5-year DFS between a high and low ALI in stage I patients (*P*=0.254; Fig. 2D). Patients with a low ALI had worse 5-year DFS than those with a high ALI in stage II (*P* = 0.031, Fig. 2E) and stage III (*P*=0.04; Fig. 2F).

### Cox regression analysis of risk factors for OS and DFS in the non-PSM cohort

In the non-PSM cohort, multivariate Cox regression analysis revealed that CA19-9 >37 U/mL (hazard ratio [HR]=1.879, *P*=0.007), pathological N+ stage (HR=3.164, *P*=0.001), LVI (HR=1.954, *P*=0.008), NI (HR=1.815, *P*=0.026), and a low ALI (HR=3.340, *P*<0.001) were independently associated with worse 5-year OS in patients who underwent CME (Supplementary Table 1).

Multivariate Cox regression analysis revealed that male sex (HR=2.156, *P*=0.002), CA19-9 >37 U/mL (HR=1.886, *P*=0.011), pathological N+ stage (HR=2.261, *P*=0.010), LVI (HR=2.285, *P* =0.002), and a low ALI (HR=2.611, *P*<0.001) were independently associated with 5-year DFS (Supplementary Table 2).

### Cox regression analysis of risk factors for OS and DFS in the PSM cohort

In the PSM cohort, multivariate Cox regression analysis revealed that age >60 years (HR=1.954, *P*=0.007), CA19-9 >37 U/mL (HR=1.964, *P*=0.011), pathological N+ stage (HR=3.266, *P*<0.001), and a low ALI (HR=3.305, *P*<0.001) were independently correlated with OS (Table 3).

**Table 3:** COX regression analysis of risk factors for overall survival of right-sided colon cancer patients in the PSM cohort

Variables	Overall survival			
	Univariate		Multivariate	
	HR (95%CI)	*P* value	HR (95%CI)	*P* value
Age (>60 vs. ≤60, years)	1.690 (1.045-2.732)	**0.032**	1.954 (1.203-3.175)	**0.007**
Gender (male vs. female)	1.281 (0.792-2.071)	0.313		
Preoperative CEA (>5 vs. ≤5, ng/ml)	1.475 (0.920-2.365)	0.106		
Preoperative CA19-9 (>37 vs. ≤37, U/ml)	2.621 (1.604-4.283)	**<0.001**	1.964 (1.168-3.303)	**0.011**
Diabetes	1.066 (0.559-2.031)	0.846		
Hypertension	1.323 (0.806-2.172)	0.268		
Tumor location (ileocecal /ascending colon vs. hepatic flexure colon)	1.287 (0.800-2.072)	0.298		
Operative time (min)	1.000 (0.995-1.005)	0.980		
Estimated blood loss (ml)	0.997 (0.993-1.002)	0.237		
pT stage (T3/4 vs. T1/2)	5.441 (0.755-39.186)	0.093	2.960(0.387-22.638)	0.296
pN stage (N+ vs. N0)	4.243 (2.478-7.267)	**<0.001**	3.266 (1.583-6.737)	**<0.001**
Tumor differentiation (grade 3+4 vs. 1+2)	1.343 (0.581-3.103)	0.490		
Histopathology (mucinous/signet ring cell adenocarcinoma vs. adenocarcinoma)	0.704 (0.427-1.164)	0.169		
Lymphovascular invasion	2.273 (1.281-4.033)	**0.005**	1.738 (0.958-3.154)	0.069
Nerval invasion	2.536 (1.407-4.569)	**0.002**	1.451 (0.776-2.710)	0.243
Postoperative complications	1.167 (0.694-1.964)	0.560		
Adjuvant chemotherapy	3.356 (1.665-6.764)	**0.001**	0.918 (0.354-2.382)	0.860
ALI (low vs. high)	3.106 (1.815-5.316)	**<0.001**	3.305 (1.927-5.667)	**<0.001**

*HR* hazard ratio, *CI* confidence interval, *ALI* advanced lung cancer inflammation index, *CEA* carcinoembryonic antigen, *CA19-9* carbohydrate antigen 19-9

In the multivariate analysis, CA19-9 >37 U/mL (HR=1.821, *P*=0.028), pathological N+ stage (HR=2.373, *P*=0.011), LVI (HR=2.271, *P*=0.005), and a low ALI (HR=2.389, *P*=0.001) remained as independent predictors of DFS (Table 4).

**Table 4:** COX regression analysis of risk factors for disease-free survival of right-sided colon cancer patients in the PSM cohort

Variables	Disease-free survival			
	Univariate		Multivariate	
	HR (95%CI)	*P* value	HR (95%CI)	*P* value
Age (>60 vs. ≤60, years)	1.370 (0.846-2.218)	0.200	1.516 (0.934-2.460)	0.092
Gender (male vs. female)	1.397 (0.854-2.283)	0.183		
Preoperative CEA (>5 vs. ≤5, ng/ml)	1.600 (0.990-2.584)	0.055		
Preoperative CA19-9 (>37 vs. ≤37, U/ml)	2.268 (1.362-3.778)	**0.002**	1.821 (1.066-3.112)	**0.028**
Diabetes	0.746 (0.357-1.562)	0.437		
Hypertension	1.303 (0.787-2.158)	0.303		
Tumor location (ileocecal /ascending colon vs. hepatic flexure colon)	1.400 (0.862-2.276)	0.174		
Operative time (min)	1.002 (0.997-1.007)	0.389		
Estimated blood loss (ml)	0.997 (0.992-1.001)	0.181		
pT stage (T3/4 vs. T1/2)	5.493 (0.762-39.579)	0.091	3.416(0.447-26.102)	0.236
pN stage (N+ vs. N0)	3.021 (1.814-5.032)	**<0.001**	2.373 (1.219-4.662)	**0.011**
Tumor differentiation (grade 3+4 vs. 1+2)	1.954 (0.933-4.089)	0.076		
Histopathology (mucinous/signet ring cell adenocarcinoma vs. adenocarcinoma)	1.257 (0.763-2.071)	0.370		
Lymphovascular invasion	2.689 (1.550-4.665)	**<0.001**	2.271 (1.289-4.001)	**0.005**
Nerval invasion	1.603 (0.818-3.142)	0.169		
Postoperative complications	0.822 (0.463-1.460)	0.503		
Adjuvant chemotherapy	2.625 (1.375-5.010)	**0.003**	0.912 (0.383-2.170)	0.835
ALI (low vs. high)	2.286 (1.372-3.806)	**0.001**	2.389 (1.434-3.982)	**0.001**

*HR* hazard ratio, *CI* confidence interval, *ALI* advanced lung cancer inflammation index, *CEA* carcinoembryonic antigen, *CA19-9* carbohydrate antigen 19-9

### Comparison of the prediction efficiency of the ALI and other parameters in the PSM cohort

**Fig. 3:**
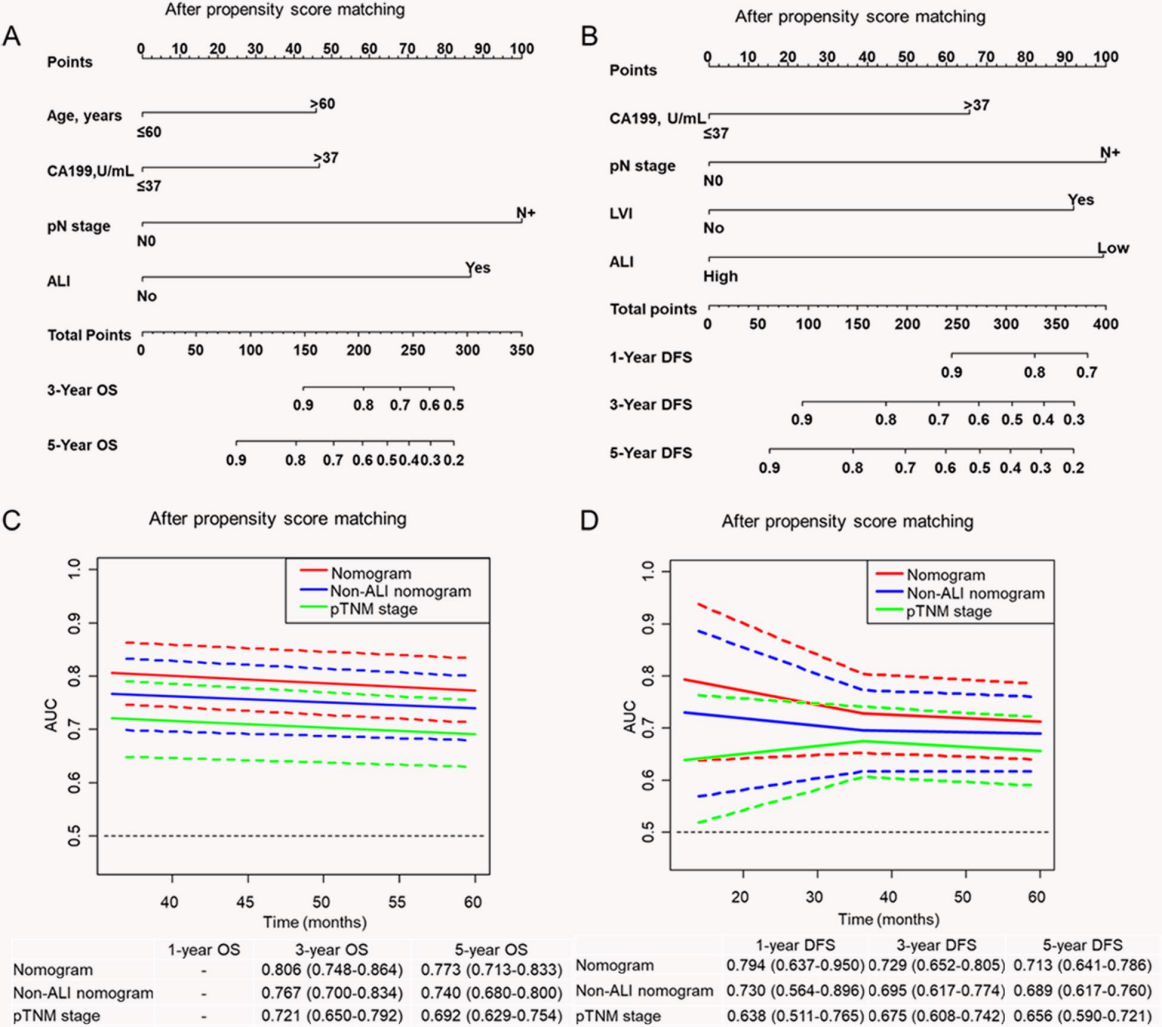
A nomogram for prediction of overall survival and disease-free survival in patients with right-sided colon cancer in the propensity-matched patients. **A** Overall survival. **B** Disease-free survival. Time-dependent ROC curves for nomogram, non-ALI nomogram, and pTNM stage in patients with right-sided colon cancer in the propensity-matched patients. **C** Overall survival. **D** Disease-free survival

Next, predictive nomograms for OS and DFS were constructed (Fig. 3A, B). The prognostic accuracy of the ALI was assessed against that of other parameters by performing a time-ROC curve analysis (Fig. 3C, D). The AUC for the nomogram combined with the ALI was optimal compared with that of the non-ALI nomogram (0.773 [95% CI 0.713–0.833]) vs. 0.740 (95% CI 0.680–0.800), *P*=0.014] and pTNM stage (0.773 [95% CI 0.713–0.833] vs. 0.692 [95% CI 0.629–0.754]; *P*<0.001) for 5-year OS (Fig. 3C). The AUC of the nomogram was better than that of the non-ALI nomogram (0.713 [95% CI 0.641–0.786]) vs. 0.689 [95% CI 0.617–0.760]; *P*=0.039) and pTNM stage (0.713 [95% CI 0.641–0.786] vs. 0.656 [95% CI 0.590–0.721]; *P*=0.002) for 5-year DFS (Fig. 3D).

In addition, the predictive ability of the nomogram combined with the ALI for 5-year OS was significantly improved compared with that of the nomogram without the ALI; its NRI increased by 24.3% (*P*=0.002) and IDI increased by 6.0% (*P*=0.002). Compared with the nomogram without the ALI, the nomogram with the ALI for 5-year OS demonstrated better predictive efficacy, its NRI increased by 32.4% (*P*<0.001), and IDI increased by 9.4% (*P*<0.001). The nomogram combined the ALI was optimal when compared with non-ALI nomogram and pTNM stage for 5-year DFS; its NRI increased by 16.8% (*P*=0.042) and 9.5% (*P*=0.042), and its IDI increased by 3.3% (*P*=0.040) and 7.5% (*P*=0.002) (Table 5).

**Table 5:** Incremental predictive value of nomogram with ALI for overall survival and disease-free survival in the PSM cohort

Variables	NRI		IDI	
	Increase value (95%CI)	*P* value	Increase value (95%CI)	*P* value
Overall survival				
Nomogram without ALI	Ref		Ref	
Nomogram with ALI	0.243 (0.104-0.364)	0.002	0.060 (0.012-0.130)	0.002
pTNM stage	Ref		Ref	
Nomogram with ALI	0.324 (0.113-0.461)	<0.001	0.094 (0.037-0.195)	<0.001
Disease-free survival				
Nomogram without ALI	Ref		Ref	
Nomogram with ALI	0.168 (0.011-0.294)	0.042	0.033 (0.002-0.083)	0.040
pTNM stage	Ref		Ref	
Nomogram with ALI	0.095 (0.003-0.339)	0.042	0.075 (0.027-0.163)	0.002

*PSM* propensity score match, *NRI* net reclassification improvement index, *IDI* integrated discrimination improvement index, *CI* confidence interval

## Discussion

Compared with left-sided colon cancer, RCC has a worse prognosis and poorer survival after recurrence [23–25]. Therefore, identifying high-risk patients with RCC after CME may aid in designing individualized treatment strategies. Currently, no studies have assessed the clinical significance of immunonutritional indicators among patients with RCC after CME. In the present study, the ALI was independently associated with short- and long-term outcomes in RCC patients after PSM analysis. In addition, the nomogram combined with the ALI demonstrated better predictive performance than that of the TNM staging system.

Inflammation and nutrition play important roles in evaluating cancer prognosis [7, 8, 26]. Nutritional and immune biomarkers in the peripheral blood, including albumin, globulin, lymphocytes, and neutrophils, are associated with the prognosis of colorectal cancer [18]. The inflammatory index can reflect the host’s immunity to cancer progression and is closely related to recurrence-free survival and OS [15]. Tumors can also affect the immune system in a pro-tumorigenic manner, increasing neutrophil and monocyte counts and decreasing lymphocyte counts [27]. Neutrophils and monocytes have been reported to be involved in cancer occurrence, growth, proliferation, and metastasis, whereas lymphocytes inhibit cancer occurrence and growth through immune surveillance [28]. Additionally, the serum albumin level is correlated with systemic inflammation during tumor proliferation and invasion, stimulates pro-inflammatory factors, and decreases albumin levels by regulating liver cell catabolism and anabolism [29, 30]. The ALI is a simple index available from routine blood tests and can be easily obtained in daily clinical practice. The correlation between the ALI and patient prognosis has been confirmed in several cancers [9–11]. However, few studies have focused on the clinical significance of the ALI among patients with RCC [12–14].

Previous studies have reported that serological inflammation-based indices (SII, NLR, and PLR) are associated with postoperative complications [31, 32]. Nutritional indicators (sarcopenia, BMI, and serum albumin level) have been used to predict postoperative complications [33–35]. In our patient cohort, most postoperative complications were infection-related. A low ALI was independently associated with postoperative morbidity. Patients with a low ALI are prone to developing postoperative complications related to poor host immune status. After PSM, the ALI remained an effective indicator for predicting postoperative complications, consistent with a previous study [14]. Together, these findings indicate that the ALI may be an effective index for predicting postoperative complications in patients with RCC. Adequate preoperative nutritional support may be beneficial reduce postoperative complications.

The ALI was independently correlated with OS and DFS compared with other combined indicators, such as PNI and the SII, owing to a complete response to systemic inflammation and the nutritional status [36]. Malnourished patients are at a higher risk for postoperative complications and, thus, have a poor prognosis after colorectal cancer surgery [37]. Pater et al. found that RCC was associated with a high tumor lymphocytic infiltrate and elevated systemic inflammation [38]. A previous study demonstrated that RCC is associated with worse prognosis than that of left-sided colon cancer [36]. Therefore, it is important to investigate the prognostic value of the ALI in those with RCC.

A low ALI is correlated with poor prognosis in many solid cancers [9–11, 33]. A low ALI score indicates impaired nutritional status and high inflammation levels. The nutritional status can also influence the host immune function [33]. Inflammation may become chronic, promote reactive oxygen and nitrogen production, and induce angiogenesis and cell proliferation, thus playing an essential role in tumorigenesis [39]. Kusunoki et al. found that a low ALI was correlated with poor OS and DFS in patients with colorectal cancer [12]. As expected, RCC patients with a low ALI had a significantly worse prognosis than those with a high ALI, particularly those in stage II and III patients. We suggest that the ALI could be used in the decision-making process when designing tailored treatments for patients.

The UICC/AJCC TNM staging system for colorectal cancer plays a significant role in evaluating treatment effects and patient prognosis [16]. However, tumor heterogeneity is common among patients with the same type of malignant tumors. A single evaluation indicator is often less sensitive in predicting prognosis, which often causes difficulties in managing patients postoperatively. Therefore, we constructed a nomogram combined with the ALI and non-ALI nomograms based on the independent risk factors in the multivariate analysis and used the time-ROC curve to compare the predictive capabilities between the pTNM stage and the two models. We found that the nomogram combined with the ALI was better than the non-ALI nomogram and TNM staging in terms of the predictive accuracy for 5-year OS and DFS. To further explore the value of the ALI in the nomogram in this study, the NRI and IDI were calculated. The NRI was used to quantify the difference in classification changes between the two models, and the IDI was used to quantify the probability difference between the two models [21, 22]. The predictive ability of the nomogram with the ALI for 5-year OS and DFS was significantly improved compared to that of the nomogram without the ALI and pTNM stage (all *P*<0.05). Therefore, the ALI can supplement the traditional TNM staging method in clinical practice to facilitate preoperative risk stratification and prognosis assessment for patients with RCC and effectively guide subsequent treatment strategies.

Postoperative adjuvant chemotherapy among stage II and III patients has been shown to improve long-term outcomes [40]. However, adjuvant chemotherapy failed to identify an independent protective factor affecting prognosis in this study. This is because high-risk stage II and III patients with RCC tend to undergo adjuvant chemotherapy [41]. When evaluating the effects of adjuvant chemotherapy, selection bias should always be considered in retrospective studies. Therefore, our study could not demonstrate a correlation between adjuvant chemotherapy and the ALI.

Postoperative surveillance aims to identify recurrence(s) and improve survival rates. However, an optimal surveillance strategy is yet to be determined. The FACS trial revealed that intensive surveillance resulted in an increased rate of surgical treatment of recurrence with curative intent [42]. Patients with a low ALI demonstrated poor 5-year OS and DFS rates. We recommend that more intensive follow-up strategies be implemented for patients with a low ALI in stage II and III; CEA monitoring and lung and abdominal CT should be performed every 3 months in the first 2 years instead of every 6 months [41]. This would be more conducive to the detection and treatment of early recurrences.

The present study had several limitations, the first of which were its retrospective design and relatively small sample size. Second, preoperative serum albumin, neutrophil, and lymphocyte levels may be influenced by many factors. Third, the cut-off values for the ALI reported in the literature vary, which may be due to the local tumor and sample sizes in previous studies, thus resulting in bias. Fourth, preoperative anemia, surgical approach, and postoperative hospital stay were statistically different between the two groups, which may have biased the results after PSM. Therefore, a prospective study is required to determine the optimal cut-off value to accurately predict the prognosis of patients with cancer.

## Conclusion

This study was the first to demonstrate that the preoperative ALI is an effective indicator for predicting short- and long-term prognosis in patients with RCC. These findings may help clinicians choose the most effective biomarkers by combining inflammation with immunity as part of individualized treatment strategies for patients with RCC, particularly those in stages II and III, to guide preoperative treatment decision-making and postoperative follow-up strategies.

## Supplementary Information

**Additional file 1: Supplementary figure 1.** The optimal cut-off value of the index in association with overall survival was determined by the X-tile software. (A) advanced lung cancer inflammation index (B) prognostic nutritional index (C) systemic inflammation index.

**Additional file 2: Supplementary figure 2.** Time-dependent ROC curves for ALI, PNI and SII in patients with right-sided colon cancer. (A) overall survival (B) disease-free survival.

**Additional file 3: Supplementary figure 3.** Kaplan-Meier survival analysis according to ALI status in patients with right sided colon cancer. (A) overall survival of patients without propensity matching patients (B) disease-free survival of patients without propensity matching.

**Additional file 4: Supplementary Table 1.** COX regression analysis of risk factors for overall survival of right-sided colon cancer patients before propensity score match.

**Additional file 5: Supplementary Table 2.** COX regression analysis of risk factors for disease-free survival of right-sided colon cancer patients before propensity score match.

## Data Availability

The data used and/or analyzed during the current study are available from the corresponding author on reasonable request.

## Abbreviations

RCC: Right-sided colon cancer
CME: Complete mesocolic excision
ALI: Advanced lung cancer inflammation index
BMI: Body mass index
ALB: Albumin
NLR: Neutrophil to lymphocyte ratio
AJCC: American Joint Committee on Cancer
TNM: Tumor-node-metastasis
SII: Systemic inflammation index
PNI: Prognostic nutritional index
NI: Nerval invasion
CEA: Carcinoembryonic antigen
CA19-9: Carbohydrate antigen 19-9
ASA: American Society of Anesthesiologists
LVI: Lymphovascular invasion
NRI: Net reclassification improvement index
IDI: Integrated discrimination improvement index
Time-ROC: Time-dependent receiver operating characteristic
OS: Overall survival
DFS: Disease-free survival
OR: Odds ratio
HR: Hazard ratio

## References

[1] Siegel RL, Miller KD, Jemal A (2020). Cancer statistics, 2020. CA Cancer J Clin.

[2] Lee GH, Malietzis G, Askari A, Bernardo D, Al-Hassi HO, Clark SK (2014). Is right-sided colon cancer different to left-sided colorectal cancer? – a systematic review. Eur J Surg Oncol.

[3] Di Buono G, Buscemi S, Cocorullo G, Sorce V, Amato G, Bonventre G (2021). Feasibility and safety of laparoscopic complete mesocolic excision (CME) for right-sided colon cancer. Ann Surg.

[4] Gao Z, Wang C, Cui Y, Shen Z, Jiang K, Shen D (2020). Efficacy and safety of complete mesocolic excision in patients with colon cancer. Ann Surg.

[5] Mahar AL, Compton C, Halabi S, Hess KR, Weiser MR, Groome PA (2017). Personalizing prognosis in colorectal cancer: a systematic review of the quality and nature of clinical prognostic tools for survival outcomes. J Surg Oncol.

[6] Colotta F, Allavena P, Sica A, Garlanda C, Mantovani A (2009). Cancer-related inflammation, the seventh hallmark of cancer: links to genetic instability. Carcinogenesis.

[7] Doleman B, Mills KT, Lim S, Zelhart MD, Gagliardi G (2016). Body mass index and colorectal cancer prognosis: a systematic review and meta-analysis. Tech Coloproctol.

[8] Gupta D, Lis CG (2010). Pretreatment serum albumin as a predictor of cancer survival: a systematic review of the epidemiological literature. Nutr J.

[9] Shiroyama T, Suzuki H, Tamiya M, Tamiya A, Tanaka A, Okamoto N (2018). Pretreatment advanced lung cancer inflammation index (ALI) for predicting early progression in nivolumab-treated patients with advanced non-small cell lung cancer. Cancer Med.

[10] Feng JF, Huang Y, Chen QX (2014). A new inflammation index is useful for patients with esophageal squamous cell carcinoma. Onco Targets Ther.

[11] Yin C, Toiyama Y, Okugawa Y, Omura Y, Kusunoki Y, Kusunoki K (2021). Clinical significance of advanced lung cancer inflammation index, a nutritional and inflammation index, in gastric cancer patients after surgical resection: a propensity score matching analysis. Clin Nutr.

[12] Kusunoki K, Toiyama Y, Okugawa Y, Yamamoto A, Omura Y, Ohi M (2020). Advanced lung cancer inflammation index predicts outcomes of patients with colorectal cancer after surgical resection. Dis Colon Rectum.

[13] Shibutani M, Maeda K, Nagahara H, Fukuoka T, Matsutani S, Kimura K (2019). The prognostic significance of the advanced lung cancer inflammation index in patients with unresectable metastatic colorectal cancer: a retrospective study. BMC Cancer.

[14] Xie H, Huang S, Yuan G, Kuang J, Yan L, Wei L (2020). The advanced lung cancer inflammation index predicts short and long-term outcomes in patients with colorectal cancer following surgical resection: a retrospective study. Peerj.

[15] Wang D, Hu X, Xiao L, Long G, Yao L, Wang Z (2021). Prognostic nutritional index and systemic immune-inflammation index predict the prognosis of patients with HCC. J Gastrointest Surg.

[16] Edge SB, Compton CC (2010). The American joint committee on cancer: the 7th edition of the AJCC cancer staging manual and the future of TNM. Ann Surg Oncol.

[17] Elinav E, Nowarski R, Thaiss CA, Hu B, Jin C, Flavell RA (2013). Inflammation-induced cancer: crosstalk between tumours, immune cells and microorganisms. Nat Rev Cancer.

[18] Sun Y, Huang Z, Lin H, Chi P (2020). Prognostic impact of preoperative immunonutritional status in rectal mucinous adenocarcinoma. Future Oncol.

[19] Hohenberger W, Weber K, Matzel K, Papadopoulos T, Merkel S (2009). Standardized surgery for colonic cancer: complete mesocolic excision and central ligation - technical notes and outcome. Color Dis.

[20] Camp RL, Dolled-Filhart M, Rimm DL (2004). X-tile: a new bio-informatics tool for biomarker assessment and outcome-based cut-point optimization. Clin Cancer Res.

[21] Burch PM, Glaab WE, Holder DJ, Phillips JA, Sauer J, Walker EG. Net reclassification index and integrated discrimination index are not appropriate for testing whether a biomarker improves predictive performance. Toxicol Sci. 2017;56:11–13. 10.1093/toxsci/kfw225 PMC5837334 27815493

[22] Pepe MS, Janes H, Li CI (2014). Net risk reclassification P values: valid or misleading?. JNCI: J Natl Cancer Inst.

[23] Fukata K, Yuasa N, Takeuchi E, Miyake H, Nagai H, Yoshioka Y (2020). Clinical and prognostic differences between surgically resected right-sided and left-sided colorectal cancer. Surg Today.

[24] Cienfuegos JA, Baixauli J, Arredondo J, Pastor C, Martinez OP, Zozaya G (2018). Clinico-pathological and oncological differences between right and left-sided colon cancer (stages I-III): analysis of 950 cases. Rev Esp Enferm Dig.

[25] Malakorn S, Ouchi A, Hu C, Sandhu L, Dasari A, You YN (2021). Tumor sidedness, recurrence, and survival after curative resection of localized colon cancer. Clin Colorectal Cancer.

[26] Hirahara N, Tajima Y, Matsubara T, Fujii Y, Kaji S, Kawabata Y (2021). Systemic immune-inflammation index predicts overall survival in patients with gastric cancer: a propensity score-matched analysis. J Gastrointest Surg.

[27] Hu Z, Wu W, Zhang X, Li P, Zhang H, Wang H (2021). Advanced lung cancer inflammation index is a prognostic factor of patients with small-cell lung cancer following surgical resection. Cancer Manag Res.

[28] Zhou S, Yuan H, Wang J, Hu X, Liu F, Zhang Y (2020). Prognostic value of systemic inflammatory marker in patients with head and neck squamous cell carcinoma undergoing surgical resection. Future Oncol.

[29] Balkwill F (2009). Tumour necrosis factor and cancer. Nat Rev Cancer.

[30] Brenner D, Blaser H, Mak TW (2015). Regulation of tumour necrosis factor signalling: live or let die. Nat Rev Immunol.

[31] Jones HG, Qasem E, Dilaver N, Egan R, Bodger O, Kokelaar R (2018). Inflammatory cell ratios predict major septic complications following rectal cancer surgery. Int J Color Dis.

[32] Sun G, Li Y, Peng Y, Lu D, Zhang F, Cui X (2019). Impact of the preoperative prognostic nutritional index on postoperative and survival outcomes in colorectal cancer patients who underwent primary tumor resection: a systematic review and meta-analysis. Int J Color Dis.

[33] Tan X, Peng H, Gu P, Chen M, Wang Y (2021). Prognostic significance of the L3 skeletal muscle index and advanced lung cancer inflammation index in elderly patients with esophageal cancer. Cancer Manag Res.

[34] De Giorgi U, Procopio G, Giannarelli D, Sabbatini R, Bearz A, Buti S (2019). Association of systemic inflammation index and body mass index with survival in patients with renal cell cancer treated with nivolumab. Clin Cancer Res.

[35] Xiao J, Caan BJ, Cespedes FE, Meyerhardt JA, Peng PD, Baracos VE (2020). Association of low muscle mass and low muscle radiodensity with morbidity and mortality for colon cancer surgery. Jama Surg.

[36] Beltran L, Gonzalez-Trejo S, Carmona-Herrera DD, Carrillo JF, Herrera-Goepfert R, Aiello-Crocifoglio V (2019). Prognostic factors and differences in survival of right and left colon carcinoma: a STROBE compliant retrospective cohort study. Arch Med Res.

[37] Parthasarathy M, Greensmith M, Bowers D, Groot-Wassink T (2017). Risk factors for anastomotic leakage after colorectal resection: a retrospective analysis of 17 518 patients. Color Dis.

[38] Patel M, McSorley ST, Park JH, Roxburgh C, Edwards J, Horgan PG (2018). The relationship between right-sided tumour location, tumour microenvironment, systemic inflammation, adjuvant therapy and survival in patients undergoing surgery for colon and rectal cancer. Br J Cancer.

[39] Mantovani A, Allavena P, Sica A, Balkwill F (2008). Cancer-related inflammation. Nature.

[40] Sadahiro S, Suzuki T, Ishikawa K, Nakamura T, Tanaka Y, Masuda T (2003). Recurrence patterns after curative resection of colorectal cancer in patients followed for a minimum of ten years. Hepatogastroenterology.

[41] Benson AB, Venook AP, Al-Hawary MM, Cederquist L, Chen YJ, Ciombor KK (2018). NCCN guidelines insights: colon cancer, version 2.2018. J Natl Compr Cancer Netw.

[42] Primrose JN, Perera R, Gray A, Rose P, Fuller A, Corkhill A (2014). Effect of 3 to 5 years of scheduled CEA and CT follow-up to detect recurrence of colorectal cancer. JAMA.

