# Supplementary figures and images for: Clinical implication of the advanced lung cancer inflammation index in patients with right-sided colon cancer after complete mesocolic excision: a propensity score-matched analysis
Source: World J Surg Oncol. 2022 Aug 1;20:246. doi: 10.1186/s12957-022-02712-0 (PMC9341074; doi:10.1186/s12957-022-02712-0)

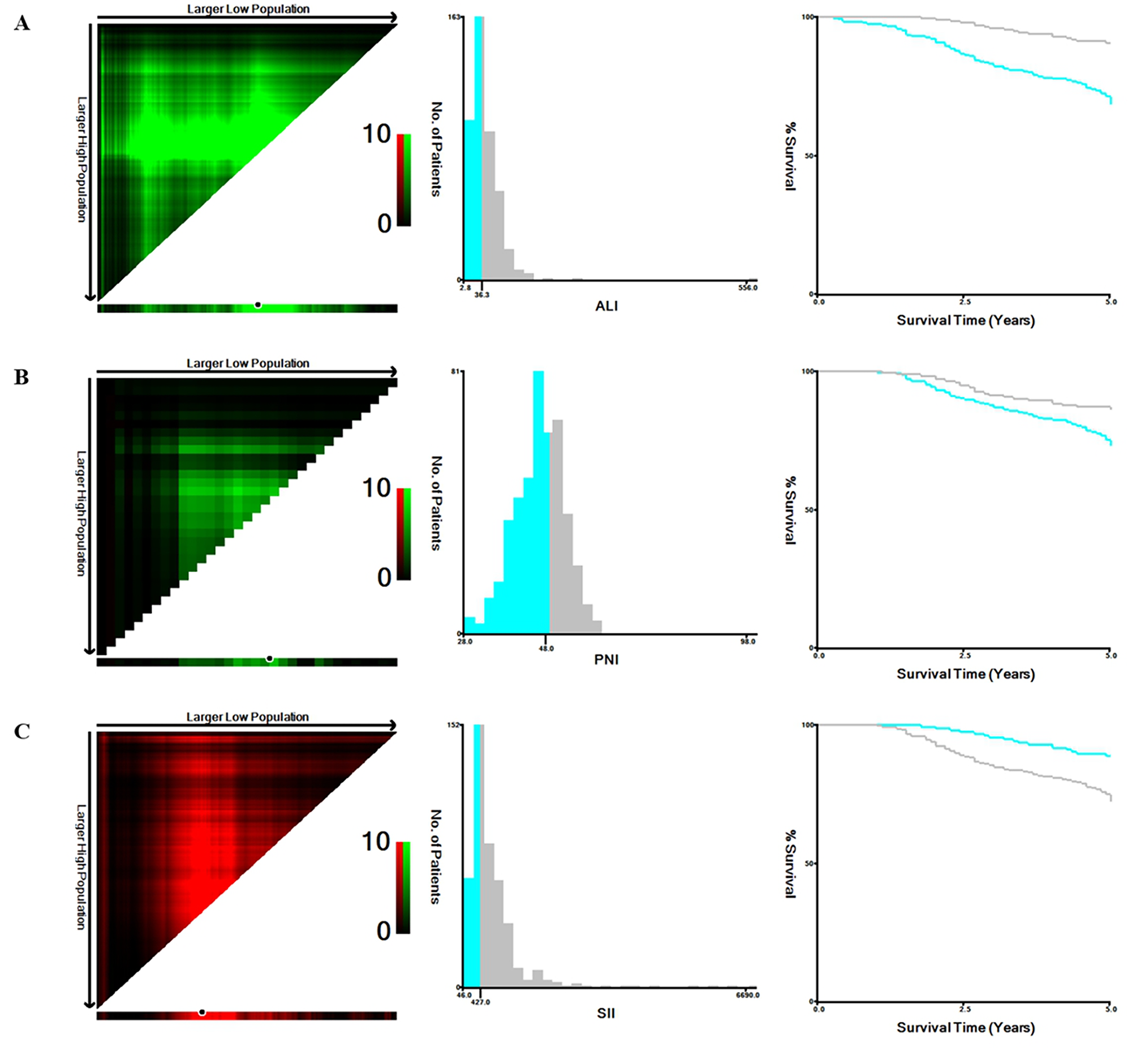

Supplement: Supplementary file 1 — Additional file 1: Supplementary figure 1. The optimal cut-off value of the index in association with overall survival was determined by the X-tile software. (A) advanced lung cancer inflammation index (B) prognostic nutritional index (C) systemic inflammation index. [file 12957_2022_2712_MOESM1_ESM.tif]

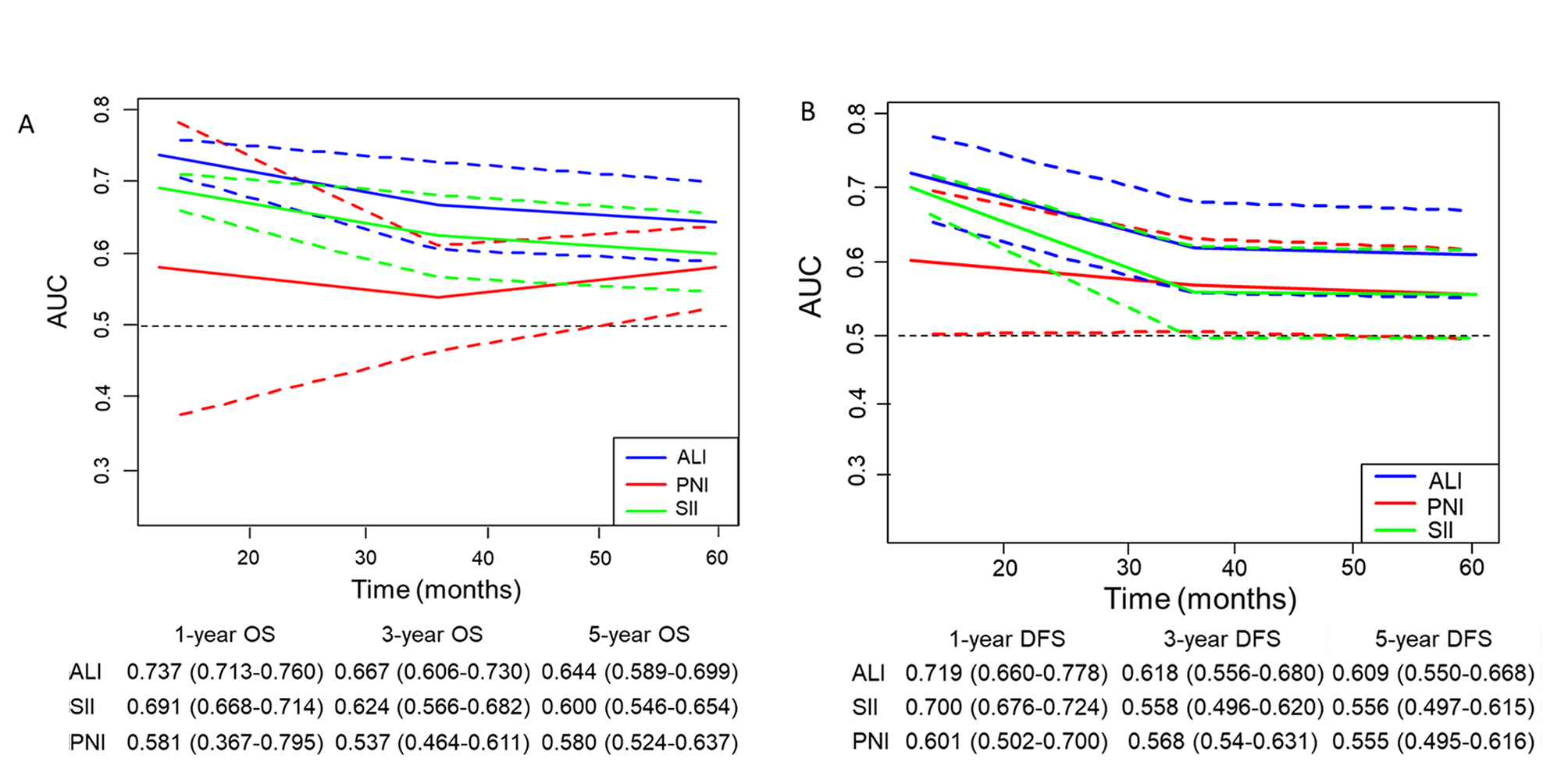

Supplement: Supplementary file 2 — Additional file 2: Supplementary figure 2. Time-dependent ROC curves for ALI, PNI and SII in patients with right-sided colon cancer. (A) overall survival (B) disease-free survival. [file 12957_2022_2712_MOESM2_ESM.tif]

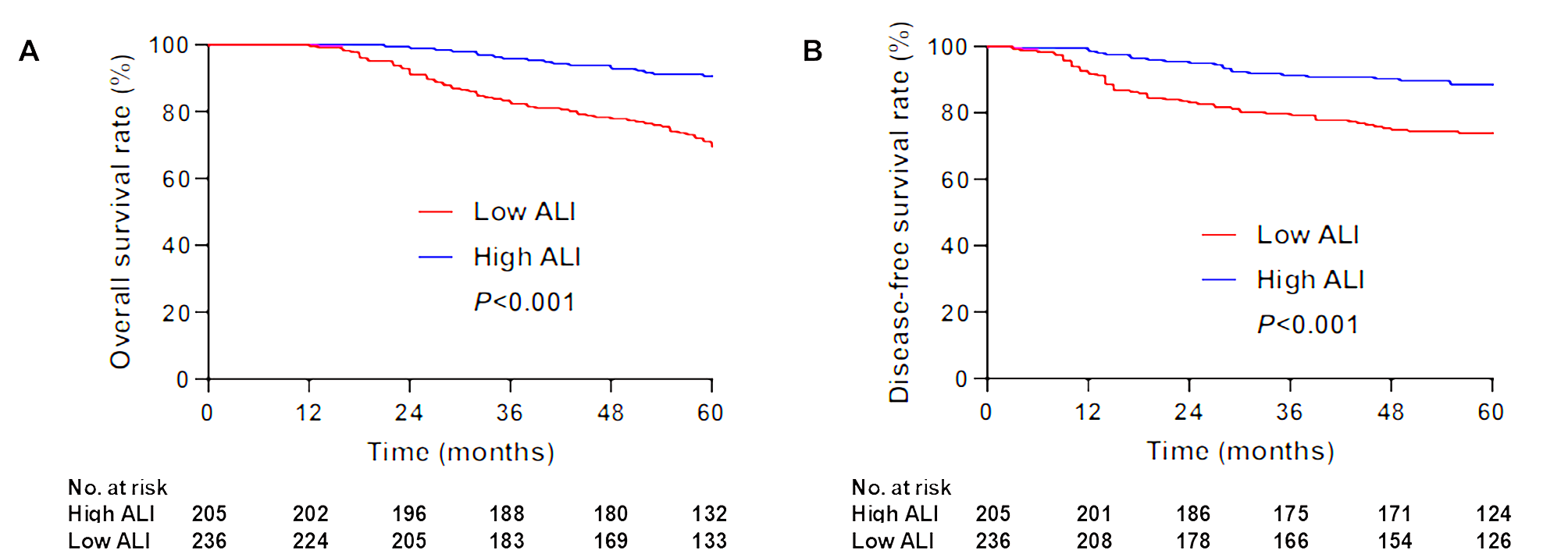

Supplement: Supplementary file 3 — Additional file 3: Supplementary figure 3. Kaplan-Meier survival analysis according to ALI status in patients with right sided colon cancer. (A) overall survival of patients without propensity matching patients (B) disease-free survival of patients without propensity matching. [file 12957_2022_2712_MOESM3_ESM.tif]
