# Supplementary material for: Clinical implication of the advanced lung cancer inflammation index in patients with right-sided colon cancer after complete mesocolic excision: a propensity score-matched analysis
Source: World J Surg Oncol. 2022 Aug 1;20:246. doi: 10.1186/s12957-022-02712-0 (PMC9341074; doi:10.1186/s12957-022-02712-0)
Supplement: Supplementary file 4 — Additional file 4: Supplementary Table 1. COX regression analysis of risk factors for overall survival of right-sided colon cancer patients before propensity score match. [file 12957_2022_2712_MOESM4_ESM.doc]

**Supplementary Table 1** COX regression analysis of risk factors for overall survival of right-sided colon cancer patients before propensity score match.

| Variables | Overall survival | | | |
| --- | --- | --- | --- | --- |
|  | Univariate | | Multivariate | |
|  | HR (95%CI) | *P* value | HR (95%CI) | *P* value |
| Age (>60 vs. ≤60, years) | 1.452 (0.937-2.249) | 0.095 |  |  |
| Gender (male vs. female) | 1.304 (0.843-2.016) | 0.233 |  |  |
| Preoperative CEA (>5 vs. ≤5, ng/ml) | 1.415 (0.922-2.172) | 0.112 |  |  |
| Preoperative CA199 (>37 vs. ≤37, U/ml) | 3.000 (1.940-4.639) | **<0.001** | 1.879 (1.191-2.965) | **0.007** |
| Diabetes | 0.933 (0.507-1.720) | 0.825 |  |  |
| Hypertension | 1.039 (0.651-1.658) | 0.873 |  |  |
| Tumor location (ileocecal/ascending colon vs. hepatic flexure colon) | 1.226 (0.798-1.885) | 0.352 |  |  |
| Operative time (min) | 1.001 (0.997-1.005) | 0.671 |  |  |
| Estimated blood loss (ml) | 0.999 (0.995-1.002) | 0.469 |  |  |
| pT stage (T3/4 vs. T1/2) | 11.254 (1.567-80.845) | **0.016** | 3.890 (0.521-29.054) | 0.185 |
| pN stage (N+ vs. N0) | 4.678 (2.830-7.732) | **<0.001** | 3.164 (1.646-6.082) | **0.001** |
| Tumor differentiation (grade 3+4 vs. 1+2) | 1.517 (0.805-2.860) | 0.198 |  |  |
| Histopathology (mucinous/signet ring cell adenocarcinoma vs. adenocarcinoma) | 0.749 (0.476-1.178) | 0.211 |  |  |
| Lymphovascular invasion | 2.968 (1.847-4.769) | **<0.001** | 1.954 (1.187-3.215) | **0.008** |
| Nerval invasion | 3.314 (2.018-5.442) | **<0.001** | 1.815 (1.074-3.069) | **0.026** |
| Postoperative complications | 1.154 (0.719-1.853) | 0.552 |  |  |
| Adjuvant chemotherapy (yes vs. no) | 4.442 (2.225-8.870) | **<0.001** | 1.015 (0.411-2.507) | 0.974 |
| ALI (low vs. high) | 3.609 (2.143-6.078) | **<0.001** | 3.340 (1.973-5.655) | **<0.001** |

HR: hazard ratio; CI: confidence interval; ALI: advanced lung cancer inflammation index; CEA: carcinoembryonic antigen; CA19-9: carbohydrate antigen 19-9;
