# Supplementary material for: Clinical implication of the advanced lung cancer inflammation index in patients with right-sided colon cancer after complete mesocolic excision: a propensity score-matched analysis
Source: World J Surg Oncol. 2022 Aug 1;20:246. doi: 10.1186/s12957-022-02712-0 (PMC9341074; doi:10.1186/s12957-022-02712-0)
Supplement: Supplementary file 5 — Additional file 5: Supplementary Table 2. COX regression analysis of risk factors for disease-free survival of right-sided colon cancer patients before propensity score match. [file 12957_2022_2712_MOESM5_ESM.doc]

**Supplementary Table 2** COX regression analysis of risk factors for disease-free survival of right-sided colon cancer patients before propensity score match.

| Variables | Disease-free survival | | | |
| --- | --- | --- | --- | --- |
|  | Univariate | | Multivariate | |
|  | HR (95%CI) | *P* value | HR (95%CI) | *P* value |
| Age (>60 vs. ≤60, years) | 1.239 (0.796-1.931) | 0.343 |  |  |
| Gender (male vs. female) | 1.701 (1.075-2.693) | **0.023** | 2.156 (1.341-3.465) | **0.002** |
| Preoperative CEA (>5 vs. ≤5, ng/ml) | 1.535 (0.988-2.386) | 0.057 |  |  |
| Preoperative CA199 (>37 vs. ≤37, U/ml) | 2.384 (1.505-3.775) | **<0.001** | 1.886 (1.157-3.075) | **0.011** |
| Diabetes | 0.700 (0.350-1.401) | 0.314 |  |  |
| Hypertension | 1.135 (0.707-1.820) | 0.601 |  |  |
| Tumor location (ileocecal/ascending colon vs. hepatic flexure colon) | 1.254 (0.807-1.948) | 0.314 |  |  |
| Operative time (min) | 1.003 (0.999-1.008) | 0.134 |  |  |
| Estimated blood loss (ml) | 0.998 (0.995-1.002) | 0.423 |  |  |
| pT stage (T3/4 vs. T1/2) | 10.950 (1.524-78.706) | **0.017** | 4.694 (0.626-35.199) | 0.133 |
| pN stage (N+ vs. N0) | 3.195 (1.990-5.128) | **<0.001** | 2.261 (1.215-4.209) | **0.010** |
| Tumor differentiation (grade 3+4 vs. 1+2) | 1.602 (0.848-3.027) | 0.147 |  |  |
| Histopathology (mucinous/signet ring cell adenocarcinoma vs. adenocarcinoma) | 0.813 (0.513-1.287) | 0.377 |  |  |
| Lymphovascular invasion | 2.887 (1.778-4.689) | **<0.001** | 2.285 (1.352-3.860) | **0.002** |
| Nerval invasion | 2.637 (1.542-4.508) | **<0.001** | 1.534 (0.857-2.746) | 0.150 |
| Postoperative complications | 0.761 (0.446-1.301) | 0.319 |  |  |
| Adjuvant chemotherapy (yes vs. no) | 3.095 (1.675-5.718) | **<0.001** | 0.974 (0.432-2.194) | 0.949 |
| ALI (low vs. high) | 2.625 (1.606-4.288) | **<0.001** | 2.611 (1.588-4.294) | **<0.001** |

HR: hazard ratio; CI: confidence interval; ALI: advanced lung cancer inflammation index; CEA: carcinoembryonic antigen; CA19-9: carbohydrate antigen 19-9
